# Altered anticipatory brain responses in eating disorders: A neuroimaging meta‐analysis

**DOI:** 10.1002/erv.2967

**Published:** 2023-01-13

**Authors:** Xinyang Yu, Sylvane Desrivières

**Affiliations:** ^1^ Social, Genetic and Developmental Psychiatry Centre Institute of Psychiatry, Psychology & Neuroscience King's College London London UK

**Keywords:** ALE meta‐analysis, anticipation, aversion, disordered eating, reward

## Abstract

**Objective:**

Functional neuroimaging studies have found differential neural activation patterns during anticipation‐related paradigms in participants with eating disorders (EDs) compared to controls. However, publications reported conflicting results on the directionality and location of the abnormal activations. There is an urgent need to integrate our existing knowledge of anticipation, both rewarding and aversive, to elucidate these differences.

**Method:**

We conducted an activation likelihood estimation (ALE) meta‐analysis to quantitatively review functional neuroimaging studies that evaluated differences between brain correlates of anticipation in participants with and without disordered eating. PubMed, Web of Sciences, PsycINFO, Medline and EMBASE were searched for studies published up to November 2022. Exploratory sub‐analyses to check for differences between reward and non‐reward anticipation among all anticipation paradigms.

**Results:**

Twenty‐one references met the inclusion criteria for meta‐analysis. The meta‐analysis across anticipation all tasks identified a significant hyperactivation cluster in the right putamen in participants with disordered eating (*n* = 17 experiments) and a significant hypoactivation cluster in the left inferior parietal lobule (*n* = 13 experiments), in participants with disordered eating compared to controls.

**Conclusions:**

These findings and sub‐analyses of reward‐ and non‐reward‐related cues suggest potential pathophysiological mechanisms underlying anticipatory responses to rewarding and aversive cues in ED.

## Key points

- The ALE meta‐analysis showed altered anticipatory brain responses in participants with disordered eating compared to controls.
- One cluster of significant hyperactivation was identified in the right putamen; another cluster of hypoactivation was found in the left inferior parietal lobule.
- Anticipatory brain responses to rewarding and aversive cues are altered in participants with disordered eating.

## 1 INTRODUCTION

The eating disorders (ED)—anorexia nervosa (AN) and bulimia nervosa (BN) are severe psychiatric illnesses prevalent in Western countries and worldwide. These conditions are characterised by negative beliefs about eating, concerns about body shape and weight, disordered eating behaviours (DEBs) including restricted eating, binge eating, and compensatory behaviours like excessive exercising, vomiting and laxative use, motivated by a drive for thinness, fear of fatness, and body image dissatisfaction (Bould et al., 2017). Eating disorders are also highly comorbid with anxiety and depression, which often co‐develop with EDs and have shared biological underpinning (Robinson et al., 2020; Zhang et al., 2020).

While their aetiology is complex, methodological advances such as functional magnetic resonance imaging (fMRI) have shed light on the neurobiology of EDs. An accumulating body of research has now clearly established an overlap between reward processing and disordered eating. Such studies suggested that alterations in the brain reward pathways underlying vulnerabilities to disordered eating (Balodis et al., 2013), might lead to abnormal anticipatory and consummatory food reward (Stice et al., 2009) and pathological eating behaviours (Morton et al., 2006). Self‐report, behavioural, and brain imaging data have revealed that obese individuals show greater anticipated food reward than lean individuals, raising the possibility that overeating may be due to hyper‐responsiveness in an anticipatory “wanting” system (Stice et al., 2009). Such anticipatory system, which involves activations of a brain circuitry of reward valuation, including the dorsal striatum (caudate and putamen), is neither directly responsible for the consummatory or hedonic experience itself (e.g., the “liking” of a palatable food or reward receipt) (Morton et al., 2006) nor a “need” or reaction to deprivation (Bosulu et al., 2022). Investigations of anticipatory and consummatory reward processes in relation to disordered eating using food (Kaye et al., 2010) or non‐food related incentive cues, such as monetary (Balodis et al., 2013), odours (Jiang et al., 2019) and touch (Wierenga et al., 2020) have generated complex findings. For example, participants with AN display diminished sensitivity to reward (Harrison et al., 2010) and diminished experience of pleasure during anticipation of food‐related cues (Soussignan et al., 2011), compared to participants with BN and healthy controls. However, neuroimaging studies using a similar food‐viewing paradigm, have reported hypoactivation either in the insula and the visual cortex (Brooks et al., 2011) or in the anterior cingulate, lateral prefrontal cortex, right middle temporal lobe and midcingulate cortex (Joos, Saum, et al., 2011) in BN compared to controls. Such inconsistencies may reflect small sample sizes, differences in study design, or sample heterogeneity. There is therefore a need to synthesise the existing knowledge to elucidate shared and distinct reward processing mechanisms in disordered eating.

Anticipation, or the expectation of a future event or stimulus, is also a hallmark of anxiety disorders, and a model integrating key psychological processes for anticipatory responses to uncertainty about future threats has been proposed to explain anxiety (Grupe & Nitschke, 2013). According to this model, alterations to these processes are responsible for maladaptive responses to uncertainty in anxious individuals, with an underlying core brain circuitry of anticipation and uncertainty, including the prefrontal cortex, striatum, and cingulate cortex, contributing to the anxious pathology. While neural mechanisms of anticipation have been at the core of a conceptual framework aimed at better understanding and treating anxiety disorders, research investigating such maladaptive anticipatory responses in EDs has been scarce. This is surprizing, given the known comorbidity between anxiety and EDs, and the fact that anxiety disorders may even predate ED onset (Kaye et al., 2004). A recent neuroimaging‐based meta‐analysis across multiple sensory modalities suggests that aversive anticipation involves brain areas implicated in reward anticipation such as the anterior cingulate (ACC), thalamus, caudate and prefrontal cortex (PFC), but fails to activate other reward‐related regions, like the nucleus accumbens (NAcc), ventral striatum and medial prefrontal cortex (mPFC; Andrzejewski et al., 2019). These findings support the existence of a universal anticipatory system, shared in reaction to reward and aversive cues. Accordingly, the exploration of both rewarding and aversive food cues in relation to EDs, has suggested that increased neural anticipatory responses, in striatal regions during anticipation of both rewarding and aversive food, and in the thalamus and amygdala during anticipation of aversive food only, might be biomarkers for AN (Cowdrey et al., 2011; Horndasch et al., 2016). However, critical questions remain as to whether these findings are robust and generalisable to other ED subtypes.

To address these questions, here we conducted the first activation likelihood estimation (ALE) meta‐analysis of whole brain fMRI studies of anticipation in disordered eating, pooling data from studies using a wide range of paradigms. Due to the lack of consensus on aversive anticipation and the inconsistent evidence of neural substrates of reward‐related anticipation, we also conducted sub‐analyses to differentiate brain responses related to reward and non‐reward anticipation in eating disorders.

## 2 METHODS

### 2.1 Search strategy and study selection

**FIGURE 1:**
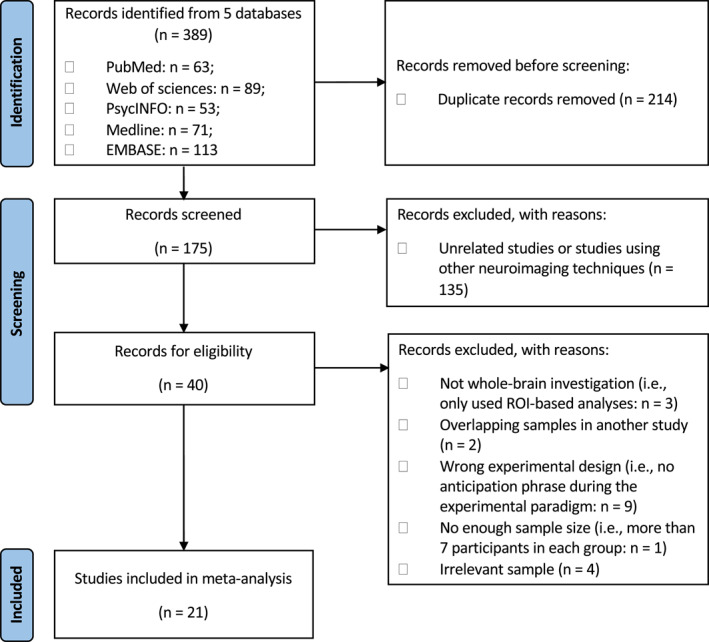
Preferred Reporting Items for Systematic and Meta‐analyses (PRISMA) flowchart detailing screening process

The review was conducted in accordance with PRISMA guidelines (Page et al., 2021), as the preferred methodology for systematic reviews and meta‐analyses. Studies were identified via electronic search in the following databases: PubMed, Web of Sciences, PsycINFO, Medline and EMBASE, with the last search date of 14 November 2022. Additionally, a manual search on the reference lists of related systematic reviews and meta‐analyses, and neuroimaging databases [NeuroSynth (neurosynth.org), BrainMap Sleuth (brainmap.org) and Brainspell (brainspell.org)] was performed to identify articles that may have been missed in the initial database search. Figure 1 is a PRISMA flow diagram describing the search for eligible studies.

Searches were conducted using combinations of key terms falling into three categories, as listed below; selected studies needed to contain at least one key term from each category.

1. Key terms of disordered eating: eating disorder, anorexia*, bulimia*, binge*, disordered eating, and eating disorders not otherwise specified (EDNOS).
2. Key terms of anticipation: anticipat*.
3. Key terms of neuroimaging techniques: fMRI, and functional magnetic resonance imaging.

All studies identified through this search were first screened by title and abstract, followed by a thorough examination of full‐text articles for final selection. Based on our research question and neuroimaging meta‐analysis guidelines (Müller et al., 2018; Tahmasian et al., 2019), studies needed to meet the following inclusion criteria.

1. To be a peer‐reviewed journal article or an abstract written in English, with full information on experimental procedures and fMRI results.
2. To include at least a participant group with disordered eating behaviours (i.e., AN or BN, eating disorders not otherwise specified, eating concerns, eating problems) and a control group. Since food addiction has emerged as a clinical entity that is recognized in the spectrum of disordered eating, for example, in patients with BN or BED (Wiss & Brewerton, 2017), we also included participants with food addiction in our analysis. Participants may consist of either current, recovered, or remitted patients.
3. To include at least 7 participants per group.
4. All study participants confirmed that they fully understood the instructions and stimuli in each task. For reward (e.g., food and money) anticipation, participants had positive emotional experiences or scored positive valence of the stimuli. For aversive anticipation, cues (i.e., aversive breathing load and pain) induced negative experiences or interoceptive taste.
5. Brain responses to reward or loss stimuli were measured by task‐activated, blood‐oxygen‐level‐dependent (BOLD) responses monitored with fMRI.
6. The experiment contained a within‐participant “reward/negative versus baseline/neutral” contrast.
7. The experiment contained a between‐group “participants with disordered eating behaviours had greater activation and/or deactivation than participants without disordered eating behaviours” contrast.
8. The field of view and reported results included the whole brain, avoiding regional selection bias and inflating results regarding the particular brain regions. If one study conducted both whole‐brain and ROI‐based analyses or small‐volume correction, we only extracted results data from whole‐brain analysis.
9. Results were adequately corrected for multiple comparisons by either reporting activation at a voxel‐level threshold of *p* < 0.001 (uncorrected) or a corrected cluster probability of *p* < 0.05.
10. Experimental results were reported in a defined stereotaxic space coordinate system [Montreal Neurological Institute (MNI) or Talairach]. Those studies that reported peak coordinates in MNI space were all converted into Talairach space before conducting the ALE meta‐analysis.

### 2.2 fMRI paradigms

Detailed descriptions the fMRI paradigms included in the analysis can be found in the Supplementary.

### 2.3 Data analysis: ALE

The meta‐analysis was performed using the revised version of the activation likelihood estimation (ALE) algorithm for coordinate‐based qualitative meta‐analyses of neuroimaging results implemented in GingerALE 3.0.2 (Eickhoff et al., 2012). Briefly, the algorithm treats activation foci reported by different studies as 3D Gaussian spatial probability distributions centred at the given coordinates and then calculates modelled activation maps of each experiment that contain the probabilities for each voxel (Turkeltaub et al., 2012). ALE scores are compared to a null distribution that assumes a random spatial association between experiments (Eickhoff et al., 2009) and combines the modelled activation maps across experiments. ALE scores indicate the convergence of results across experiments at each particular location in the brain.

In line with recent guidelines based on massive ALE simulations (Eickhoff et al., 2016), the resulting non‐parametric *p* values were then thresholded at a cluster‐level family‐wise error (FWE) corrected threshold of *p* < 0.05 (cluster‐forming threshold at voxel‐level *p* < 0.001) and *N* = 1000 permutations.

Sub‐analyses: Food‐addicted participants were primarily included in the analysis, since the concept of food addiction refers to excessive and dysregulated consumption of high‐energy food (Imperatori et al., 2016), and is highly correlated to binge eating (Leigh & Morris, 2018), which is well in line with our rationale for disordered eating. However, considering that food addiction may impact the reward circuitry like drug addiction, thereby distorting our results, we performed an additional analysis after excluding the two food addiction‐related studies. We also conducted exploratory sub‐analyses to check for differences between reward and non‐reward anticipation among all anticipation paradigms.

### 2.4 Sensitivity analysis

#### 2.4.1 Fail‐Safe N (FSN) analysis

The publication bias is defined as the problem that the selective submission and publication of statistically significant over negative studies, and may result in potential overestimating effects and inaccurate representation of executed research (Acar et al., 2018; Dickersin et al., 1987; Kicinski, 2014). Fail‐Safe N (FSN) quantifies the number of null studies (studies with negative results or contra‐evidence) that are needed to change current results (Acar et al., 2018; Rosenthal, 1979). In an ALE meta‐analysis, the FSN can be obtained for each cluster that survives thresholding in the analysis. We used the R programme (https://github.com/NeuroStat/GenerateNull) to calculate the FSN for each cluster. We calculated the FSN for activation and deactivation cluster among those anticipation paradigms to evaluate the robustness of our results and compare current FSNs with the minimal likelihood of publication bias [5*k* + 10, with *k* referring to the number of studies; suggested by Rosenthal (1979)], with a larger number indicating higher robustness.

#### 2.4.2 Jackknife analysis

The Jackknife analysis provided another alternative to evaluate the sensitivity and to which extent the results could be replicated. To assess the impact of each single study on the results, we ran a whole‐brain voxel‐based jackknife sensitivity analysis (with the leave‐one‐out method). Specifically, we computed meta‐analyses of activation results for 17 times simulations, and deactivation results for 14 times simulations, respectively, with a single different experiment of the original sample being left out each time. Same as the initial ALE analysis, we also used an FWE correction and 1000 permutations to define significant results. Then, we inspected how well each of these simulations reproduced the initial results in terms of number, peak coordinate location and size of significant ALE cluster size.

## 3 RESULTS

### 3.1 Study selection

A total of 21 studies were eligible for inclusion in the meta‐analysis (Figure 1 and Table 1). Almost all of them investigated AN, BN and BED (19 studies; including current, remitted and recovered patients), and only two included participants with food addiction.

**TABLE 1:** Studies included in the meta‐analysis

Study	Participants with disordered eating (DE)						Participants without DE			Task	Stimuli type	Contrast/condition
	*N*	Type	M/F	Age	Comorbidity	Medication	*N*	M/F	Age			
Balodis et al. (2013)	19	BED	5/14	43.7	1 had mood disorder and 1 had anxiety disorder	Sibutramine and cognitive behavioural‐self‐help interventions	19	9/10	34.8	Monetary incentive delay task	Money	Win versus no win anticipation
												Win versus no win receipt
												Loss versus no loss anticipation
												Loss versus no loss receipt
Berner et al. (2018)	17	Women remitted from AN	0/17	26.5			25	0/25	28.3	Aversive inspiratory breathing load paradigm	Breathing load	Breathing load anticipation
												Breathing load
												Post‐breathing load
Berner et al. (2019)	24	Women remitted from BN	0/24	27.5			25	0/25	25.2	Aversive inspiratory breathing load paradigm	Breathing load	Breathing load anticipation
												Breathing load
Bischoff‐Grethe et al. (2018)	18	Women remitted from AN	0/18	26.3			26	0/26	26.3	Soft touch paradigm	Touch	Touch anticipation
												Soft touch receipt
Brooks et al. (2011)	8	BN	0/8	25.0			24	0/24	26	Food images viewing paradigm	Food	Food versus non‐food anticipation
	18	AN	0/18	26.0								
Cowdrey et al. (2011)	15	Recovered AN	0/15	23.3	4 had bulimia nervosa and 1 had eating disorder not otherwise specified;		16	0/16	24.1	Pleasant chocolate and aversive strawberry paradigm	Food	Pleasant food anticipation
												Pleasant food taste
												Aversive food anticipation
					9 had major depressive disorder (MDD) and 3 had obsessive‐compulsive disorder							Aversive food taste
Ehrlich et al. (2015)	30	Recovered patients	0/30	22.0			30	0/30	21.6	Instrumental motivation task	Money	Reward anticipation
												Motor response
												Feedback
Gearhardt et al. (2011)	15	High food addiction	0/15	NG			11	0/11	NG	Milkshake paradigm	Food	Food anticipation
												Food receipt
Horndasch et al. (2018)	15	AN adolescents	0/15	16.4		Antidepressant, antipsychotic, and antiepileptic medication	18	0/18	15.95	Food picture viewing paradigm	Food	High calorie anticipation
	16	AN adult	0/16	26.7			16	0/16	26.88			Low calorie anticipation
Jiang et al. (2019)	14	Restrictive AN	0/14	24.9			12	0/12	24.14	Odour liking and wanting task	Food odour	Food odour liking
	13	BN	0/13	22.5								Food odour wanting
Joos, Saum, et al. (2011)	13	BN	0/13	25.2		Citalopram	13	0/13	27.0	Food paradigm	Food	Food versus non‐food anticipation
Kaye et al. (2010)	14	Recovered AN	0/14	NG			14	0/14	NG	Picture viewing paradigm	Food	Food anticipation
	14	Recovered BN	0/14	NG							Neutral	Neutral objects anticipation
Murao et al. (2017)	11	Restricting‐type AN	0/11	30.9	Some AN participants had comorbid symptoms, such as anxiety, depressive mood, aggression, and/or impulsiveness	Combinations of psychoactive medications (i.e., antipsychotics, antidepressants, antianxiolytics, and antiepileptics)	20	0/20	33.2	Monetary incentive delay task	Money	Win versus no win anticipation
	12	Binge eating/purging‐type AN	0/12	39.3								Loss versus no loss anticipation
Oberndorfer et al. (2013)	13	Recovered AN	0/13	26.0			11	0/11	28.9	Visual anticipatory task	Food	Food anticipation
												Food receipt
Scaife et al. (2016)	14	Recovered AN	0/14	27.0	2 AN had depression, 4 AN had depression and generalised anxiety disorder	Antidepressant and atypical antipsychotic	16	0/16	24.3	Picture viewing paradigm	Food	High calorie anticipation
	12	AN patient	0/12	19.4								Low calorie anticipation
Schulte et al. (2019)	20	High food addiction	0/20	29.9	2 for overweight and 17 for obesity		24	0/24	31.08	Food cure reactivity paradigm	Food	Food anticipation
Simon et al. (2016)	27	BED	NG	38.3		Antidepressant medication	28	NG	38.0	Monetary incentive delay task	Money	High versus no reward anticipation
												High versus no reward receipt
												High versus no food reward anticipation
	29	BN		27.5			27		25.7	Food incentive delay task	Food	High versus no food reward anticipation
Strigo et al. (2013)	12	Recovered AN	0/12	29.7			12	0/12	24.8	Experimental pain paradigm	Pain	Pain anticipation
												Pain stimulation
Uher et al. (2003)	9	Recovered AN	0/9	26.9		Antidepressant medication (SSRI)	9	0/9	26.6	Picture viewing paradigm	Food	Food versus non‐food anticipation
	8	Chronic AN	0/8	25.6								
Uher et al. (2004)	26	16 AN and 10BN	0/26	27.9		Antidepressant medication (selective serotonin reuptake inhibitors)	19	0/19	26.7	Food and aversive images paradigm	Food and aversive image	Food versus non‐food anticipation
												Aversive versus neutral anticipation
Wierenga et al. (2020)	23	Remitted from BN	0/23	25.6			25	0/25	27.2	Soft touch paradigm	Touch	Soft touch anticipation

Abbreviations: AN, anorexia nervosa; BN, bulimia nervosa; BED, binge eating disorder; NG, not given, but matched between patient groups and healthy controls.

### 3.2 Study characteristics

All participants with disordered eating met either one of the following criteria: (1) Current patients: those who had a current ED diagnosis based on a structured, semi‐structured or clinical interview for Diagnostic and Statistical Manual of Mental Disorders (DSM‐IV) (American Psychiatric Association, 1994), DSM‐V (American Psychiatric Association, 2013) or International Statistical Classification of Diseases and Related Health problems (ICD‐10) criteria of BN or AN, including restricting AN and binge purging AN, and those recruited from the inpatient and outpatient services of hospitals; (2) Remitted or recovered patients: those who had a past ED diagnosis (i.e., no ED diagnoses for at least 12 months before the study); (3) Recovered patients: patients who maintained a weight above 85% average body weight; had regular menstrual cycles and did not binge, purged, or engaged in significant restrictive eating patterns for at least 6 months before the study; (4) Participants with high food addiction: these met the criteria for moderate‐to‐severe food addition based on the Yale Food Addiction Scale (YFAS 2.0) with three or more symptoms based on the DSM‐5 criteria.

Participants without disordered eating had no history of any ED or psychiatric disorder and were in the normal weight range or without food addiction measured by YFAS scale (with one or fewer symptoms).

### 3.3 Activation likelihood estimation (ALE) analyses of anticipatory responses across all paradigms

**FIGURE 2:**
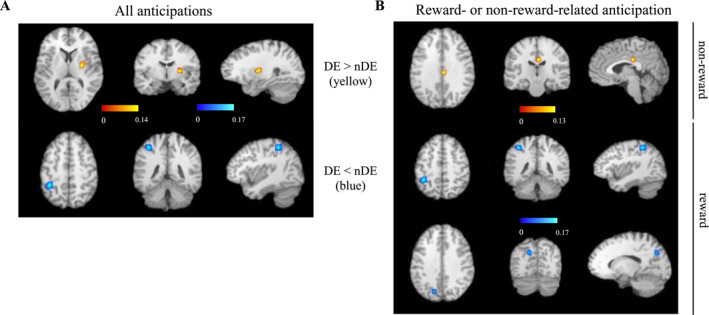
ALE maps of clusters showing group differences in activation during all types of anticipation (a) and reward‐ and non reward‐related anticipation tasks (b). Yellow labels indicate clusters with higher activations in participants with disordered eating (DE) compared to controls (nDE). Blue labels indicate clusters with lower activations in participants with DE compared to controls. The peak coordinates for the activation and deactivation clusters centred in A, at (26, −8, 6; activation, right putamen) and (−36, −44, 48; deactivation, left IPL) and in B, at (4, −24, 28; activation, right cingulate gyrus), (−36, −44, 48; deactivation, left IPL) and (−14, −72, 32; deactivation, left cuneus/precuneus)

Analysis of anticipatory BOLD signals across all anticipation paradigms revealed significant clusters of convergent differences when comparing groups of participants with and without disordered eating (Table 2 and Figure 2a). A significant cluster of activation (cluster size = 664, *z* = 4.36; *n* = 17 experiments) had two peaks primarily located in the right putamen (95.7%), extending into the lateral globus pallidus (1.4%). One significant cluster of convergent deactivations (cluster size = 920, *z* = 5.36; *n* = 13 experiments) was observed, with a single peak in the left inferior parietal lobule (IPL; 97.8%), extending into the superior parietal lobule (2.2%).

**TABLE 2:** Brain regions and Talairach coordinates for peaks showing convergence in activation (DE > nDE) or deactivation (DE < nDE) during all and reward or non‐reward anticipation tasks

Condition	Cluster's anatomical location (BA)	Talairach coordinates			ALE (×10^−3^)	*Z* values	*p* values	Cluster size (mm^3^)
		*x*	*y*	*z*				
All anticipation								
DE > nDE	Right putamen	26	−8	6	13.9	4.37	6.14E‐06	664
		26	−12	8	13.5	4.28		
DE < nDE	Left inferior parietal lobule (40)	−36	−44	48	16.8	5.36	4.06E‐08	920
Reward anticipation								
DE < nDE	Left inferior parietal lobule (40)	−36	−44	48	16.8	5.62	9.42E‐09	976
	Left cuneus (7)/precuneus	−14	−72	32	12.2	4.59	2.27E‐06	472
*Non‐reward anticipation*								
DE > nDE	Right cingulate gyrus (23)	4	−24	28	12.4	4.57	2.43E‐06	440

Two studies with food addicted subjects were included in our analyses. They contributed only to analyses of anticipatory brain activations and may therefore have distorted our finding of a significant cluster of activation in the right putamen. Therefore, we re‐run our analyses after excluding these two studies, which did not affect our results; the cluster of activation in the right putamen remained significant (cluster size = 672, z = 4.40, *n* = 15 experiments; Supplementary Table S1 and Figure S1), indicating that our findings were not distorted by food addiction.

### 3.4 Sensitivity analyses

We performed two types of sensitivity analyses to test the robustness of these findings. FSN analyses indicated that for the significant cluster of activation in the right putamen, the number of null studies required to change these results was over 180, exceeding the minimum of 95 for 17 experiments. These results indicated a robust convergence of foci in the right putamen during anticipatory brain activation. Jackknife sensitivity analysis, which simulates the effects of removing one study at a time on the overall finding, indicated that this activation cluster remained significant in almost all repetitions (i.e., 15 out of 17 simulations; Supplementary Table S2). Two experiments contributed most to the findings, reporting at least one foci within this activation cluster. These studies reported higher activations in this region in remitted BN during anticipation of aversive inspiratory breathing load (Berner et al., 2019) and in recovered AN during anticipation of food images (Oberndorfer et al., 2013), compared to controls.

For the significant cluster of deactivation in the left IPL, FSN analyses indicated that the number of null studies required to change our results was over 150, exceeding the minimum number of 80 for 14 experiments. This result supports a robust convergence of foci in this region. In the Jackknife sensitivity analysis, this cluster remained significant in 13 of the 14 simulations (Supplementary Table S3). Three experiments contributed most to the findings, with at least one foci with this deactivation cluster. These reported lower activation within this region in ED patients compared to healthy controls during food anticipation paradigms. More specifically, in current and recovered AN during anticipation of low‐calorie food (Scaife et al., 2016), and in recovered AN patients (Uher et al., 2003) and ED patients (AN and BN; Uher et al., 2004) in food versus non‐food anticipation.

### 3.5 Sub‐analyses of reward‐ and non‐reward‐related anticipation

To specifically test for potential contributions of rewarding versus non‐rewarding stimuli to ED psychopathology, we repeated our ALE analyses in reward and non‐reward related fMRI tasks separately. In the analyses across all reward anticipation paradigms comparing participants with and without disordered eating (*n* = 11 experiments), the initial activation cluster in the right putamen was not found to be significant and no other significant activation cluster was observed. In contrast, two significant clusters of convergent deactivations (*n* = 10 experiments) were identified (Table 2 and Figure 2b). The first (cluster size = 976, *z* = 5.62), primarily in the left IPL (98%) and extending into the left superior parietal lobule (2%), reproduced the findings from anticipatory deactivation analyses across all studies reported above. The second (cluster size = 472, *z* = 4.59) was in the left cuneus (55.2%), extending into left precuneus (44.8%). Two experiments contributed at least one focus on the deactivation cluster in this region. These studies used food anticipation paradigms to compare recovered AN (Uher et al., 2003) or subjects with current ED (AN and BN; Uher et al., 2004) to healthy controls.

Analysis of non‐reward‐related anticipation (Table 2 and Figure 2b) revealed one cluster of convergent activation (cluster size = 440, *z* = 4.57; *n* = 5 experiments) in the right cingulate gyrus (100%) when comparing participants with and without disordered eating. Two experiments related to anticipation of aversive stimuli significantly contributed to the results. One compared recovered BN to healthy controls during anticipation of aversive breathing (Berner et al., 2019), and the other compared recovered AN to healthy controls during pain anticipation (Strigo et al., 2013). However, given the low number of experiments, these results should be interpreted with great caution.

## 4 DISCUSSION

In this first ALE meta‐analysis of fMRI anticipatory brain responses in participants with disordered eating, we found that compared to healthy controls, participants with a history of ED (AN or BN) displayed higher activation in the right putamen during anticipation of positive and negative affective cues across multiple paradigms. Sub‐analyses of reward‐ and non‐reward‐related cues further revealed higher activation in the right cingulate gyrus in participants with a history of ED, specifically during aversive anticipation. Anticipatory responses in ED participants were also characterised by hypoactivation in the left IPL, an effect that was driven by reward‐related (i.e., food) cues. Sub‐analyses of reward‐related anticipation also revealed the left precuneus/cuneus as hypoactivated in these participants in reactivity to food. Although the fMRI studies included in our analyses are diverse, our results lend support to the existence of alterations in universal and specific anticipatory systems in the pathophysiology of ED.

Our finding of increased activation in the right putamen across anticipation paradigms, including positive and negative affective cues, supports previous suggestions of the existence of a universal anticipatory system, comprising striatal regions, that becomes activated in response to both rewarding and aversive stimuli (Cowdrey et al., 2011; Horndasch et al., 2016). The putamen may be part of a core circuit for long‐term memory‐based stable reward value discrimination (Kunimatsu et al., 2019). Further, the putamen, along with other components of the basal forebrain and the cingulate gyrus, has been identified as part of a right‐lateralised circuit critical for expressing preparatory fear responses, and activated during sustained rather than immediate aversive anticipatory activity (Grupe et al., 2013). This is well in line with the role of the dorsal striatum (caudate and putamen) in stimulus‐response learning or habit learning (Packard & Knowlton, 2002), including aversive learning (Stanley et al., 2021). Our findings suggest maladaptive functioning of this circuit in ED. Accordingly, a recent study suggested that putamen activity may reflect changes in monitoring reward value in binge eating disorders (Hartogsveld et al., 2022). Of note, larger grey matter volumes in the right putamen/globus pallidus were found to predate the development of binge eating behaviours (Zhang et al., 2020). Finally, the striatum plays a role in reward and motivation by mediating dopamine responses (Schultz, 1998). A study combining fMRI and positron emission tomography in humans supported this view further, correlating neural activation in the striatum with striatal dopamine release during reward anticipation (Schott et al., 2008). Thus, the convergent hyperactivity in the putamen found in participants with disordered eating, might reflect an altered dopaminergic transmission.

On the contrary, two cortical regions, the left IPL—a brain region consistently activated during anticipation of positive rewards in healthy subjects (Liu et al., 2011)—and the left cuneus/precuneus, were hypoactivated in ED in response to reward‐related stimuli. Although limited experiments warrant caution in interpreting the cuneus/precuneus findings, these results are concordant with several studies highlighting a critical role for these two regions in the pathophysiology of AN. Structural neuroimaging studies have consistently reported lower GMV (Castro‐Fornieles et al., 2009; Gaudio et al., 2011; Joos, Hartmann, et al., 2011; Joos et al., 2010) and a thinner cortex (de la Cruz et al., 2021) in the IPL and precuneus of AN patients compared to controls. We note that differences present during acute phases of the disease (Castro‐Fornieles et al., 2009; Gaudio et al., 2011; Joos et al., 2010) were found to persist into recovery (Joos, Hartmann, et al., 2011). This is in line with our findings linking hypoactivation of these brain regions to studies of food anticipation in current and recovered AN (Scaife et al., 2016; Uher et al., 2003, 2004). Intriguingly, these two regions involved in visuospatial memory and imagery have been proposed to be responsible for the perceptive component of the body image distortion in AN (Gaudio & Quattrocchi, 2012). Beyond ED, the left IPL has been proposed as a potential predictive biomarker for PTSD treatment response in a study (van Rooij et al., 2015) that reported increased pre‐treatment activation of this region in treatment responders compared with non‐responders during the stop‐signal anticipation task. Whether this region is also a predictive biomarker for ED remains to be investigated.

As pointed out above, regions identified in our study were also altered in recovered participants, making them potential areas involved in the etiopathogenesis of ED. The right lateralised hyperactivations in the putamen and cingulate gyrus were observed in both recovered BN (Berner et al., 2019) and recovered AN (Oberndorfer et al., 2013; Strigo et al., 2013), while the left lateralised hypoactivations in the IPL and precuneus/cuneus seemed to be more specifically related to AN recovery (Scaife et al., 2016; Uher et al., 2003). However, longitudinal analyses will be needed to unambiguously determine whether these neural markers represent traits or scars of the disease.

Several limitations should be noted in the present study. First, only 21 papers were included in this meta‐analysis, limiting the interpretation of our results. Such limited studies might also explain why we could not find changes in activation in other expected brain areas, such as the amygdala and anterior cingulate cortex, whose involvement in anticipation are well‐documented. This also limited the extent of our sub‐analyses. For example, we were unable to differentiate between types of disordered eating (i.e., anorexia nervosa, bulimia nervosa, binge eating disorder and food addiction) and status of eating disorder patients (i.e., current patients and recovered/remitted patients), which may increase the heterogeneity of the current results. At last, the ALE meta‐analysis can only quantify convergence probabilities but not the magnitude of activations of those significant clusters.

## 5 CONCLUSION

The neural substrates of EDs are starting to be elucidated but many unanswered questions remain. Despite the importance of anticipatory brain responses to reward and aversion, their disturbances in ED main poorly investigated. This first meta‐analytic synthesis of research on anticipation in ED identified two distinct clusters of significant activation and deactivation distinguishing participants with and without disordered eating, providing clues about the pathophysiology of ED. Yet, to fully advance our understanding of these disorders with complex presentations, more studies are needed. In particular, studies comparing ED subtypes will be required to clarify activation patterns that are shared and distinct across ED subtypes, or tasks, and those that depend on the type of stimuli.

## Supporting information

Supplementary Material

## Data Availability

Data sharing is not applicable to this article as no new data were created or analyzed in this study.

## References

[erv2967-bib-0001] Acar, F. , Seurinck, R. , Eickhoff, S. B. , & Moerkerke, B. (2018). Assessing robustness against potential publication bias in Activation Likelihood Estimation (ALE) meta‐analyses for fMRI. PLoS One, 13(11), e0208177. 10.1371/journal.pone.0208177 30500854 PMC6267999

[erv2967-bib-0002] American Psychiatric Association . (1994). Diagnostic and statistical manual of mental health disorders (4th ed.). American Psychiatric Publishing.

[erv2967-bib-0003] American Psychiatric Association . (2013). Diagnostic and statistical manual of mental disorders. American Psychiatric Publishing.

[erv2967-bib-0004] Andrzejewski, J. A. , Greenberg, T. , & Carlson, J. M. (2019). Neural correlates of aversive anticipation: An activation likelihood estimate meta‐analysis across multiple sensory modalities. Cognitive, Affective, & Behavioral Neuroscience, 19(6), 1379–1390. 10.3758/s13415-019-00747-7 31502205

[erv2967-bib-0005] Balodis, I. M. , Kober, H. , Worhunsky, P. D. , White, M. A. , Stevens, M. C. , Pearlson, G. D. , Sinha, R. , Grilo, C. M. , & Potenza, M. N. (2013). Monetary reward processing in obese individuals with and without binge eating disorder. Biological Psychiatry, 73(9), 877–886. 10.1016/j.biopsych.2013.01.014 23462319 PMC3686098

[erv2967-bib-0006] Berner, L. A. , Simmons, A. N. , Wierenga, C. E. , Bischoff‐Grethe, A. , Paulus, M. P. , Bailer, U. , Ely, A. V. , & Kaye, W. H. (2018). Altered interoceptive activation before, during, and after aversive breathing load in women remitted from anorexia nervosa. Psychological Medicine, 48(1), 142–154. 10.1017/S0033291717001635 28714434 PMC5990016

[erv2967-bib-0007] Berner, L. A. , Simmons, A. N. , Wierenga, C. E. , Bischoff‐Grethe, A. , Paulus, M. P. , Bailer, U. F. , & Kaye, W. H. (2019). Altered anticipation and processing of aversive interoceptive experience among women remitted from bulimia nervosa. Neuropsychopharmacology, 44(7), 1265–1273. 10.1038/s41386-019-0361-4 30840983 PMC6785154

[erv2967-bib-0008] Bischoff‐Grethe, A. , Wierenga, C. E. , Berner, L. A. , Simmons, A. N. , Bailer, U. , Paulus, M. P. , & Kaye, W. H. (2018). Neural hypersensitivity to pleasant touch in women remitted from anorexia nervosa. Translational Psychiatry, 8(1), 161. 10.1038/s41398-018-0218-3 30115929 PMC6095886

[erv2967-bib-0009] Bosulu, J. , Allaire, M.‐A. , Tremblay‐Grénier, L. , Luo, Y. , Eickhoff, S. , & Hétu, S. (2022). ‘Wanting’ versus ‘needing’ related value: An fMRI meta‐analysis. Brain and Behavior, 12(9), e32713. 10.1002/brb3.2713 36000558 PMC9480935

[erv2967-bib-0010] Bould, H. , Newbegin, C. , Stewart, A. , Stein, A. , & Fazel, M. (2017). Eating disorders in children and young people. BMJ, 359, j5245. 10.1136/bmj.j5245 29217505

[erv2967-bib-0011] Brooks, S. J. , O′Daly, O. G. , Uher, R. , Friederich, H.‐C. , Giampietro, V. , Brammer, M. , Williams, S. C. R. , Schiöth, H. B. , Treasure, J. , & Campbell, I. C. (2011). Differential neural responses to food images in women with bulimia versus anorexia nervosa. PLoS One, 6(7), e22259. 10.1371/journal.pone.0022259 21799807 PMC3140495

[erv2967-bib-0013] Castro‐Fornieles, J. , Bargalló, N. , Lázaro, L. , Andrés, S. , Falcon, C. , Plana, M. T. , & Junqué, C. (2009). A cross‐sectional and follow‐up voxel‐based morphometric MRI study in adolescent anorexia nervosa. Journal of Psychiatric Research, 43(3), 331–340. 10.1016/j.jpsychires.2008.03.013 18486147

[erv2967-bib-0014] Cowdrey, F. A. , Park, R. J. , Harmer, C. J. , & McCabe, C. (2011). Increased neural processing of rewarding and aversive food stimuli in recovered anorexia nervosa. Biological Psychiatry, 70(8), 736–743. 10.1016/j.biopsych.2011.05.028 21714958

[erv2967-bib-0015] de la Cruz, F. , Schumann, A. , Suttkus, S. , Helbing, N. , Zopf, R. , & Bär, K.‐J. (2021). Cortical thinning and associated connectivity changes in patients with anorexia nervosa. Translational Psychiatry, 11(1), 95. 10.1038/s41398-021-01237-6 33542197 PMC7862305

[erv2967-bib-0016] Dickersin, K. , Chan, S. , Chalmersx, T. C. , Sacks, H. S. , & Smith, H. (1987). Publication bias and clinical trials. Controlled Clinical Trials, 8(4), 343–353. 10.1016/0197-2456(87)90155-3 3442991

[erv2967-bib-0017] Ehrlich, S. , Geisler, D. , Ritschel, F. , King, J. A. , Seidel, M. , Boehm, I. , Breier, M. , Clas, S. , Weiss, J. , Marxen, M. , Smolka, M. N. , Roessner, V. , & Kroemer, N. B. (2015). Elevated cognitive control over reward processing in recovered female patients with anorexia nervosa. Journal of Psychiatry & Neuroscience: JPN, 40(5), 307–315. 10.1503/jpn.140249 26107161 PMC4543093

[erv2967-bib-0018] Eickhoff, S. B. , Bzdok, D. , Laird, A. R. , Kurth, F. , & Fox, P. T. (2012). Activation likelihood estimation meta‐analysis revisited. NeuroImage, 59(3), 2349–2361. 10.1016/j.neuroimage.2011.09.017 21963913 PMC3254820

[erv2967-bib-0019] Eickhoff, S. B. , Laird, A. R. , Grefkes, C. , Wang, L. E. , Zilles, K. , & Fox, P. T. (2009). Coordinate‐based activation likelihood estimation meta‐analysis of neuroimaging data: A random‐effects approach based on empirical estimates of spatial uncertainty. Human Brain Mapping, 30(9), 2907–2926. 10.1002/hbm.20718 19172646 PMC2872071

[erv2967-bib-0020] Eickhoff, S. B. , Nichols, T. E. , Laird, A. R. , Hoffstaedter, F. , Amunts, K. , Fox, P. T. , Bzdok, D. , & Eickhoff, C. R. (2016). Behavior, sensitivity, and power of activation likelihood estimation characterized by massive empirical simulation. NeuroImage, 137, 70–85. 10.1016/j.neuroimage.2016.04.072 27179606 PMC4981641

[erv2967-bib-0021] Gaudio, S. , Nocchi, F. , Franchin, T. , Genovese, E. , Cannatà, V. , Longo, D. , & Fariello, G. (2011). Gray matter decrease distribution in the early stages of Anorexia Nervosa restrictive type in adolescents. Psychiatry Research, 191(1), 24–30. 10.1016/j.pscychresns.2010.06.007 21081268

[erv2967-bib-0022] Gaudio, S. , & Quattrocchi, C. C. (2012). Neural basis of a multidimensional model of body image distortion in anorexia nervosa. Neuroscience & Biobehavioral Reviews, 36(8), 1839–1847. 10.1016/j.neubiorev.2012.05.003 22613629

[erv2967-bib-0023] Gearhardt, A. N. , Yokum, S. , Orr, P. T. , Stice, E. , Corbin, W. R. , & Brownell, K. D. (2011). The neural correlates of “food addiction”. Archives of General Psychiatry, 68(8), 808–816. 10.1001/archgenpsychiatry.2011.32 21464344 PMC3980851

[erv2967-bib-0024] Grupe, D. W. , & Nitschke, J. B. (2013). Uncertainty and anticipation in anxiety. Nature Reviews Neuroscience, 14(7), 488–501. 10.1038/nrn3524 23783199 PMC4276319

[erv2967-bib-0025] Grupe, D. W. , Oathes, D. J. , & Nitschke, J. B. (2013). Dissecting the anticipation of aversion reveals dissociable neural networks. Cerebral Cortex, 23(8), 1874–1883. 10.1093/cercor/bhs175 22763169 PMC3698367

[erv2967-bib-0026] Harrison, A. , O’Brien, N. , Lopez, C. , & Treasure, J. (2010). Sensitivity to reward and punishment in eating disorders. Psychiatry Research, 177(1), 1–11. 10.1016/j.psychres.2009.06.010 20381877

[erv2967-bib-0027] Hartogsveld, B. , Quaedflieg, C. W. E. M. , van Ruitenbeek, P. , & Smeets, T. (2022). Decreased putamen activation in balancing goal‐directed and habitual behavior in binge eating disorder. Psychoneuroendocrinology, 136, 105596. 10.1016/j.psyneuen.2021.105596 34839081

[erv2967-bib-0028] Horndasch, S. , O’Keefe, S. , Lamond, A. , Brown, K. , & McCabe, C. (2016). Increased anticipatory but decreased consummatory brain responses to food in sisters of anorexia nervosa patients. BJPsych Open, 2(4), 255–261. 10.1192/bjpo.bp.115.002550 27703784 PMC4995168

[erv2967-bib-0029] Horndasch, S. , Roesch, J. , Forster, C. , Dörfler, A. , Lindsiepe, S. , Heinrich, H. , Graap, H. , Moll, G. H. , & Kratz, O. (2018). Neural processing of food and emotional stimuli in adolescent and adult anorexia nervosa patients. PLoS One, 13(3), e0191059. 10.1371/journal.pone.0191059 29579064 PMC5868769

[erv2967-bib-0030] Imperatori, C. , Fabbricatore, M. , Vumbaca, V. , Innamorati, M. , Contardi, A. , & Farina, B. (2016). Food addiction: Definition, measurement and prevalence in healthy subjects and in patients with eating disorders. Rivista di Psichiatria, 51(2), 60–65. 10.1708/2246.24196 27183510

[erv2967-bib-0031] Jiang, T. , Soussignan, R. , Carrier, E. , & Royet, J.‐P. (2019). Dysfunction of the mesolimbic circuit to food odors in women with anorexia and bulimia nervosa: A fMRI study. Frontiers in Human Neuroscience, 13, 117. 10.3389/fnhum.2019.00117 31019456 PMC6458263

[erv2967-bib-0033] Joos, A. , Hartmann, A. , Glauche, V. , Perlov, E. , Unterbrink, T. , Saum, B. , Tüscher, O. , Tebartz van Elst, L. , & Zeeck, A. (2011). Grey matter deficit in long‐term recovered anorexia nervosa patients. European Eating Disorders Review: The Journal of the Eating Disorders Association, 19(1), 59–63. 10.1002/erv.1060 21038322

[erv2967-bib-0034] Joos, A. , Klöppel, S. , Hartmann, A. , Glauche, V. , Tüscher, O. , Perlov, E. , Saum, B. , Freyer, T. , Zeeck, A. , & Tebartz van Elst, L. (2010). Voxel‐based morphometry in eating disorders: Correlation of psychopathology with grey matter volume. Psychiatry Research, 182(2), 146–151. 10.1016/j.pscychresns.2010.02.004 20400273

[erv2967-bib-0035] Joos, A. A. B. , Saum, B. , Zeeck, A. , Perlov, E. , Glauche, V. , Hartmann, A. , Freyer, T. , Sandholz, A. , Unterbrink, T. , Tebartz van Elst, L. , & Tüscher, O. (2011). Frontocingular dysfunction in bulimia nervosa when confronted with disease‐specific stimuli. European Eating Disorders Review, 19(5), 447–453. 10.1002/erv.1150 21809423

[erv2967-bib-0036] Kaye, W. H. , Bulik, C. M. , Thornton, L. , Barbarich, N. , & Masters, K. , & The Price Foundation Collaborative Group . (2004). Comorbidity of anxiety disorders with anorexia and bulimia nervosa. American Journal of Psychiatry, 161(12), 2215–2221. 10.1176/appi.ajp.161.12.2215 15569892

[erv2967-bib-0037] Kaye, W. H. , Oberndorfer, T. , Frank, G. , Fudge, J. L. , Simmons, A. , Wagner, A. , Paulus, M. , & Grethe, A. (2010). Insula response to sweet taste and anticipation of food in anorexia and bulimia nervosa: Is altered sensory‐interoceptive function a biomarker that reflects the urge to eat? Neuropsychopharmacology, 35(1), S23. 10.1038/npp.2010.215

[erv2967-bib-0038] Kicinski, M. (2014). How does under‐reporting of negative and inconclusive results affect the false‐positive rate in meta‐analysis? A simulation study. BMJ Open, 4(8), e004831. 10.1136/bmjopen-2014-004831 PMC4156818 25168036

[erv2967-bib-0039] Kunimatsu, J. , Maeda, K. , & Hikosaka, O. (2019). The caudal part of putamen represents the historical object value information. Journal of Neuroscience: The Official Journal of the Society for Neuroscience, 39(9), 1709–1718. 10.1523/JNEUROSCI.2534-18.2018 30573645 PMC6391567

[erv2967-bib-0040] Leigh, S.‐J. , & Morris, M. J. (2018). The role of reward circuitry and food addiction in the obesity epidemic: An update. Biological Psychology, 131, 31–42. 10.1016/j.biopsycho.2016.12.013 28011401

[erv2967-bib-0041] Liu, X. , Hairston, J. , Schrier, M. , & Fan, J. (2011). Common and distinct networks underlying reward valence and processing stages: A meta‐analysis of functional neuroimaging studies. Neuroscience & Biobehavioral Reviews, 35(5), 1219–1236. 10.1016/j.neubiorev.2010.12.012 21185861 PMC3395003

[erv2967-bib-0042] Morton, G. J. , Cummings, D. E. , Baskin, D. G. , Barsh, G. S. , & Schwartz, M. W. (2006). Central nervous system control of food intake and body weight. Nature, 443(7109), 289–295. 10.1038/nature05026 16988703

[erv2967-bib-0043] Müller, V. I. , Cieslik, E. C. , Laird, A. R. , Fox, P. T. , Radua, J. , Mataix‐Cols, D. , Tench, C. R. , Yarkoni, T. , Nichols, T. E. , Turkeltaub, P. E. , Wager, T. D. , & Eickhoff, S. B. (2018). Ten simple rules for neuroimaging meta‐analysis. Neuroscience & Biobehavioral Reviews, 84, 151–161. 10.1016/j.neubiorev.2017.11.012 29180258 PMC5918306

[erv2967-bib-0044] Murao, E. , Sugihara, G. , Isobe, M. , Noda, T. , Kawabata, M. , Matsukawa, N. , Takahashi, H. , Murai, T. , & Noma, S. (2017). Differences in neural responses to reward and punishment processing between anorexia nervosa subtypes: An fMRI study. Psychiatry and Clinical Neurosciences, 71(9), 647–658. 10.1111/pcn.12537 28459134

[erv2967-bib-0045] Oberndorfer, T. , Simmons, A. , McCurdy, D. , Strigo, I. , Matthews, S. , Yang, T. , Irvine, Z. , & Kaye, W. (2013). Greater anterior insula activation during anticipation of food images in women recovered from anorexia nervosa versus controls. Psychiatry Research, 214(2), 132‐141. 10.1016/j.pscychresns.2013.06.010 23993362 PMC3880160

[erv2967-bib-0046] Packard, M. G. , & Knowlton, B. J. (2002). Learning and memory functions of the basal Ganglia. Annual Review of Neuroscience, 25(1), 563–593. 10.1146/annurev.neuro.25.112701.142937 12052921

[erv2967-bib-0047] Page, M. J. , McKenzie, J. E. , Bossuyt, P. M. , Boutron, I. , Hoffmann, T. C. , Mulrow, C. D. , Shamseer, L. , Tetzlaff, J. M. , Akl, E. A. , Brennan, S. E. , Chou, R. , Glanville, J. , Grimshaw, J. M. , Hróbjartsson, A. , Lalu, M. M. , Li, T. , Loder, E. W. , Mayo‐Wilson, E. , McDonald, S. , … Moher, D. (2021). The PRISMA 2020 statement: An updated guideline for reporting systematic reviews. BMJ, 372, n71. 10.1136/bmj.n71 33782057 PMC8005924

[erv2967-bib-0048] Robinson, L. , Zhang, Z. , Jia, T. , Bobou, M. , Roach, A. , Campbell, I. , Irish, M. , Quinlan, E. B. , Tay, N. , Barker, E. D. , Banaschewski, T. , Bokde, A. L. W. , Grigis, A. , Garavan, H. , Heinz, A. , Ittermann, B. , Martinot, J.‐L. , Stringaris, A. , Penttilä, J. , … IMAGEN Consortium . (2020). Association of genetic and phenotypic assessments with onset of disordered eating behaviors and comorbid mental health problems among adolescents. JAMA Network Open, 3(12), e2026874. 10.1001/jamanetworkopen.2020.26874 33263759 PMC7711322

[erv2967-bib-0049] Rosenthal, R. (1979). The file drawer problem and tolerance for null results. Psychological Bulletin, 86(3), 638–641. 10.1037/0033-2909.86.3.638

[erv2967-bib-0050] Scaife, J. C. , Godier, L. R. , Reinecke, A. , Harmer, C. J. , & Park, R. J. (2016). Differential activation of the frontal pole to high vs low calorie foods: The neural basis of food preference in Anorexia Nervosa? Psychiatry Research: Neuroimaging, 258, 44–53. 10.1016/j.pscychresns.2016.10.004 27866012 PMC5146322

[erv2967-bib-0051] Schott, B. H. , Minuzzi, L. , Krebs, R. M. , Elmenhorst, D. , Lang, M. , Winz, O. H. , Seidenbecher, C. I. , Coenen, H. H. , Heinze, H.‐J. , Zilles, K. , Düzel, E. , & Bauer, A. (2008). Mesolimbic functional magnetic resonance imaging activations during reward anticipation correlate with reward‐related ventral striatal dopamine release. Journal of Neuroscience, 28(52), 14311–14319. 10.1523/JNEUROSCI.2058-08.2008 19109512 PMC6671462

[erv2967-bib-0052] Schulte, E. M. , Yokum, S. , Jahn, A. , & Gearhardt, A. N. (2019). Food cue reactivity in food addiction: A functional magnetic resonance imaging study. Physiology & Behavior, 208, 112574. 10.1016/j.physbeh.2019.112574 31181233 PMC6620138

[erv2967-bib-0053] Schultz, W. (1998). Predictive reward signal of dopamine neurons. Journal of Neurophysiology, 80(1), 1–27. 10.1152/jn.1998.80.1.1 9658025

[erv2967-bib-0054] Simon, J. J. , Skunde, M. , Walther, S. , Bendszus, M. , Herzog, W. , & Friederich, H.‐C. (2016). Neural signature of food reward processing in bulimic‐type eating disorders. Social Cognitive and Affective Neuroscience, 11(9), 1393–1401. 10.1093/scan/nsw049 27056455 PMC5015798

[erv2967-bib-0055] Soussignan, R. , Schaal, B. , Rigaud, D. , Royet, J.‐P. , & Jiang, T. (2011). Hedonic reactivity to visual and olfactory cues: Rapid facial electromyographic reactions are altered in anorexia nervosa. Biological Psychology, 86(3), 265–272. 10.1016/j.biopsycho.2010.12.007 21185351

[erv2967-bib-0056] Stanley, A. T. , Lippiello, P. , Sulzer, D. , & Miniaci, M. C. (2021). Roles for the dorsal striatum in aversive behavior. Frontiers in Cellular Neuroscience, 15, 634493. 10.3389/fncel.2021.634493 33664651 PMC7920955

[erv2967-bib-0057] Stice, E. , Spoor, S. , Ng, J. , & Zald, D. H. (2009). Relation of obesity to consummatory and anticipatory food reward. Physiology & Behavior, 97(5), 551–560. 10.1016/j.physbeh.2009.03.020 19328819 PMC2734415

[erv2967-bib-0058] Strigo, I. A. , Matthews, S. C. , Simmons, A. N. , Oberndorfer, T. , Klabunde, M. , Reinhardt, L. E. , & Kaye, W. H. (2013). Altered insula activation during pain anticipation in individuals recovered from anorexia nervosa: Evidence of interoceptive dysregulation. International Journal of Eating Disorders, 46(1), 23–33. 10.1002/eat.22045 22836447 PMC3507323

[erv2967-bib-0059] Tahmasian, M. , Sepehry, A. A. , Samea, F. , Khodadadifar, T. , Soltaninejad, Z. , Javaheripour, N. , Khazaie, H. , Zarei, M. , Eickhoff, S. B. , & Eickhoff, C. R. (2019). Practical recommendations to conduct a neuroimaging meta‐analysis for neuropsychiatric disorders. Human Brain Mapping, 40(17), 5142–5154. 10.1002/hbm.24746 31379049 PMC6865620

[erv2967-bib-0060] Turkeltaub, P. E. , Eickhoff, S. B. , Laird, A. R. , Fox, M. , Wiener, M. , & Fox, P. (2012). Minimizing within‐experiment and within‐group effects in activation likelihood estimation meta‐analyses. Human Brain Mapping, 33(1), 1–13. 10.1002/hbm.21186 21305667 PMC4791073

[erv2967-bib-0061] Uher, R. , Brammer, M. J. , Murphy, T. , Campbell, I. C. , Ng, V. W. , Williams, S. C. R. , & Treasure, J. (2003). Recovery and chronicity in anorexia nervosa: Brain activity associated with differential outcomes. Biological Psychiatry, 54(9), 934–942. 10.1016/S0006-3223(03)00172-0 14573322

[erv2967-bib-0062] Uher, R. , Murphy, T. , Brammer, M. J. , Dalgleish, T. , Phillips, M. L. , Ng, V. W. , Andrew, C. M. , Williams, S. C. R. , Campbell, I. C. , & Treasure, J. (2004). Medial prefrontal cortex activity associated with symptom provocation in eating disorders. American Journal of Psychiatry, 161(7), 1238–1246. 10.1176/appi.ajp.161.7.1238 15229057

[erv2967-bib-0063] van Rooij, S. J. H. , Geuze, E. , Kennis, M. , Rademaker, A. R. , & Vink, M. (2015). Neural correlates of inhibition and contextual cue processing related to treatment response in PTSD. Neuropsychopharmacology: Official Publication of the American College of Neuropsychopharmacology, 40(3), 667–675. 10.1038/npp.2014.220 25154707 PMC4289955

[erv2967-bib-0064] Wierenga, C. E. , Bischoff‐Grethe, A. , Berner, L. A. , Simmons, A. N. , Bailer, U. , Paulus, M. P. , & Kaye, W. H. (2020). Increased anticipatory brain response to pleasant touch in women remitted from bulimia nervosa. Translational Psychiatry, 10(1), 236. 10.1038/s41398-020-00916-0 32669557 PMC7363900

[erv2967-bib-0065] Wiss, D. A. , & Brewerton, T. D. (2017). Incorporating food addiction into disordered eating: The disordered eating food addiction nutrition guide (DEFANG). Eating and Weight Disorders, 22(1), 49–59. 10.1007/s40519-016-0344-y 27943202 PMC5334442

[erv2967-bib-0066] Zhang, Z. , Robinson, L. , Jia, T. , Quinlan, E. B. , Tay, N. , Chu, C. , Barker, E. D. , Banaschewski, T. , Barker, G. J. , Bokde, A. L. W. , Flor, H. , Grigis, A. , Garavan, H. , Gowland, P. , Heinz, A. , Ittermann, B. , Martinot, J.‐L. , Stringaris, A. , Penttilä, J. , … Desrivières, S. (2020). Development of disordered eating behaviors and comorbid depressive symptoms in adolescence: Neural and psychopathological predictors. Biological Psychiatry, 90(12), 853–862. S0006322320316723. 10.1016/j.biopsych.2020.06.003 32778392

