# Supplementary material for: Altered anticipatory brain responses in eating disorders: A neuroimaging meta‐analysis
Source: Eur Eat Disord Rev. 2023 Jan 13;31(3):363–76. doi: 10.1002/erv.2967 (PMC10947459; doi:10.1002/erv.2967)

**Supplementary**

[fMRI anticipation paradigms 1](#_Toc119342357)

[References 4](#_Toc119342358)

[Table S1. 8](#_Toc119342359)

[Table S2. 9](#_Toc119342360)

[Table S3. 10](#_Toc119342361)

[Figure S1. 11](#_Toc119342362)

# fMRI anticipation paradigms

The monetary incentive delay (MID) task is a widely used and validated reward processing task adapted for use in human fMRI studies. Each trial consists of an anticipation phase and a receipt phase: in the anticipation phase, participants are given a cue signalling the potential win or loss of a monetary reward, or neutral stimuli signalling no amount of money; then they are instructed to press a button to ‘win’ the reward; in the receipt phase, participants will receive feedback on win or loss of money and also view their cumulative earning on the task (Balodis et al., 2013; Murao et al., 2017).

The food incentive delay (FID) task is an adaptive version of the original MID task that was found to reliably induce activity in brain regions related to the anticipation and receipt of food rewards. In the FID paradigm, an empty basket, or a small, or a large basket filled with snacks are used as a reward, corresponding to the no ‘win’, small or large monetary reward in MID, respectively. Here again, each trial includes an anticipation phase and a receipt phase (Simon et al., 2016).

The instrumental motivation task, another modified version of MID, takes advantage of behavioural assessment of motivation operationalized as instrumental responding to maximize reward (Bühler et al., 2010). Interindividual performance differences in motor speed are standardized in pretest runs which measure maximum motor responses (maximum number of button presses). Each trial includes an anticipation phase, a motor response phase and a receipt phase (Ehrlich et al., 2015).

During the food viewing task/visual anticipatory task, participants are instructed to view standardized images of food and non-food neutral valence items (household). Participants are then instructed on one of the following: (1) to rate their pleasantness, intensity and wanting for the stimuli (Cowdrey et al., 2011); (2) to report the pleasantness/unpleasantness rating of the images (Oberndorfer et al., 2013); (3) to imagine eating the food in the images or using the non-food items (Brooks et al., 2011); (4) to look at each picture attentively or explicitly and rate it for emotional experience or valence (Horndasch et al., 2018; Joos et al., 2011); (5) to think about how much they wanted each item presented, as if it were in front of them (Scaife et al., 2016; Schulte et al., 2019); (6) to look at each image and think what it makes them feel like (Uher et al., 2003, 2004). These instructions allow for the measurement of brain activation during the period of anticipatory processing/anticipated consumption and/or consumption processing.

The milkshake task examines the activation in response to anticipated consumption and consumption of palatable food. Artificial saliva and chocolate milkshake are used as neutral and palatable stimuli in the task, respectively. Pictures, such as a glass of milkshake or a glass of water, are presented prior to participants as a cue, followed by tastes delivered several seconds later (actual food receipt) in 40% of trials (Gearhardt et al., 2011).

The aversive inspiratory breathing load task is used to study aversive interoception by inducing a negative interoceptive state, which also avoids the confounds of symptom-specific responses to food stimuli in eating disorder patients. During the task, participants are instructed to breathe through a hose that intermittently restricted breathing for a cued period, and coloured shapes are used to signal the likelihood of an upcoming breathing load period. Each trial included an anticipation phase, a breathing load phase and a post-breathing load phase (Berner et al., 2018, 2019).

The soft-touch task is used to measure the mismatch between an anticipated and experienced outcome that may contribute to maladaptive behaviours in psychiatric patients, including recovered BN patients (Wierenga et al., 2020). Arrows in different directions indicated whether absence or presence and the likelihood of a subsequent soft touch. Each trial included an anticipation phase and a receipt (experience a soft touch) phase (Bischoff-Grethe et al., 2018).

The odour wanting and liking task operationalizes the motivation of a food reward as two distinct components, namely, the ‘wanting’ and the ‘liking’ components. During the task, food odours are utilized to induce affective responses: participants are instructed to rate their desire to eat the food in wanting trials, and respond to their liking judgment in liking trials. Each session included a ‘odour wanting’ (anticipation) run and a ‘odour liking’ (receipt) run (Jiang et al., 2015, 2019).

The experimental pain task utilizes high or low thermal pain stimuli to produce moderate and mild pain sensations. Crosses in different colours are signalled to ‘high pain’, ‘low pain’ or ‘unknown’ stimuli with different probabilities and are presented to participants to cue anticipation of varying pain levels during the task. Each session included a pain anticipation condition and a pain stimulation condition (Strigo et al., 2013).

# References

Balodis, I. M., Kober, H., Worhunsky, P. D., White, M. A., Stevens, M. C., Pearlson, G. D., Sinha, R., Grilo, C. M., & Potenza, M. N. (2013). Monetary Reward Processing in Obese Individuals With and Without Binge Eating Disorder. *Biological Psychiatry*, *73*(9), 877–886. https://doi.org/10.1016/j.biopsych.2013.01.014

Berner, L. A., Simmons, A. N., Wierenga, C. E., Bischoff-Grethe, A., Paulus, M. P., Bailer, U., Ely, A. V., & Kaye, W. H. (2018). Altered Interoceptive Activation Before, During, and After Aversive Breathing Load in Women Remitted from Anorexia Nervosa. *Psychological Medicine*, *48*(1), 142–154. https://doi.org/10.1017/S0033291717001635

Berner, L. A., Simmons, A. N., Wierenga, C. E., Bischoff-Grethe, A., Paulus, M. P., Bailer, U. F., & Kaye, W. H. (2019). Altered anticipation and processing of aversive interoceptive experience among women remitted from bulimia nervosa. *Neuropsychopharmacology*, *44*(7), 1265–1273. https://doi.org/10.1038/s41386-019-0361-4

Bischoff-Grethe, A., Wierenga, C. E., Berner, L. A., Simmons, A. N., Bailer, U., Paulus, M. P., & Kaye, W. H. (2018). Neural hypersensitivity to pleasant touch in women remitted from anorexia nervosa. *Translational Psychiatry*, *8*, 161. https://doi.org/10.1038/s41398-018-0218-3

Brooks, S. J., O′Daly, O. G., Uher, R., Friederich, H.-C., Giampietro, V., Brammer, M., Williams, S. C. R., Schiöth, H. B., Treasure, J., & Campbell, I. C. (2011). Differential Neural Responses to Food Images in Women with Bulimia versus Anorexia Nervosa. *PLoS ONE*, *6*(7), e22259. https://doi.org/10.1371/journal.pone.0022259

Bühler, M., Vollstädt-Klein, S., Kobiella, A., Budde, H., Reed, L. J., Braus, D. F., Büchel, C., & Smolka, M. N. (2010). Nicotine Dependence Is Characterized by Disordered Reward Processing in a Network Driving Motivation. *Biological Psychiatry*, *67*(8), 745–752. https://doi.org/10.1016/j.biopsych.2009.10.029

Cowdrey, F. A., Park, R. J., Harmer, C. J., & McCabe, C. (2011). Increased Neural Processing of Rewarding and Aversive Food Stimuli in Recovered Anorexia Nervosa. *Biological Psychiatry*, *70*(8), 736–743. https://doi.org/10.1016/j.biopsych.2011.05.028

Ehrlich, S., Geisler, D., Ritschel, F., King, J. A., Seidel, M., Boehm, I., Breier, M., Clas, S., Weiss, J., Marxen, M., Smolka, M. N., Roessner, V., & Kroemer, N. B. (2015). Elevated cognitive control over reward processing in recovered female patients with anorexia nervosa. *Journal of Psychiatry & Neuroscience : JPN*, *40*(5), 307–315. https://doi.org/10.1503/jpn.140249

Gearhardt, A. N., Yokum, S., Orr, P. T., Stice, E., Corbin, W. R., & Brownell, K. D. (2011). The Neural Correlates of “Food Addiction”. *Archives of General Psychiatry*, *68*(8), 808–816. https://doi.org/10.1001/archgenpsychiatry.2011.32

Horndasch, S., Roesch, J., Forster, C., Dörfler, A., Lindsiepe, S., Heinrich, H., Graap, H., Moll, G. H., & Kratz, O. (2018). Neural processing of food and emotional stimuli in adolescent and adult anorexia nervosa patients. *PLOS ONE*, *13*(3), e0191059. https://doi.org/10.1371/journal.pone.0191059

Jiang, T., Soussignan, R., Carrier, E., & Royet, J.-P. (2019). Dysfunction of the Mesolimbic Circuit to Food Odors in Women With Anorexia and Bulimia Nervosa: A fMRI Study. *Frontiers in Human Neuroscience*, *13*, 117. https://doi.org/10.3389/fnhum.2019.00117

Jiang, T., Soussignan, R., Schaal, B., & Royet, J.-P. (2015). Reward for food odors: An fMRI study of liking and wanting as a function of metabolic state and BMI. *Social Cognitive and Affective Neuroscience*, *10*(4), 561–568. https://doi.org/10.1093/scan/nsu086

Joos, A. A. B., Saum, B., Zeeck, A., Perlov, E., Glauche, V., Hartmann, A., Freyer, T., Sandholz, A., Unterbrink, T., Elst, L. T. van, & Tüscher, O. (2011). Frontocingular Dysfunction in Bulimia Nervosa when Confronted with Disease-specific Stimuli. *European Eating Disorders Review*, *19*(5), 447–453. https://doi.org/10.1002/erv.1150

Murao, E., Sugihara, G., Isobe, M., Noda, T., Kawabata, M., Matsukawa, N., Takahashi, H., Murai, T., & Noma, S. (2017). Differences in neural responses to reward and punishment processing between anorexia nervosa subtypes: An fMRI study. *Psychiatry and Clinical Neurosciences*, *71*(9), 647–658. https://doi.org/10.1111/pcn.12537

Oberndorfer, T., Simmons, A., McCurdy, D., Strigo, I., Matthews, S., Yang, T., Irvine, Z., & Kaye, W. (2013). Greater anterior insula activation during anticipation of food images in women recovered from anorexia nervosa versus controls. *Psychiatry Research*, *214*(2), 10.1016/j.pscychresns.2013.06.010. https://doi.org/10.1016/j.pscychresns.2013.06.010

Scaife, J. C., Godier, L. R., Reinecke, A., Harmer, C. J., & Park, R. J. (2016). Differential activation of the frontal pole to high vs low calorie foods: The neural basis of food preference in Anorexia Nervosa? *Psychiatry Research: Neuroimaging*, *258*, 44–53. https://doi.org/10.1016/j.pscychresns.2016.10.004

Schulte, E. M., Yokum, S., Jahn, A., & Gearhardt, A. N. (2019). Food cue reactivity in food addiction: A functional magnetic resonance imaging study. *Physiology & Behavior*, *208*, 112574. https://doi.org/10.1016/j.physbeh.2019.112574

Simon, J. J., Skunde, M., Walther, S., Bendszus, M., Herzog, W., & Friederich, H.-C. (2016). Neural signature of food reward processing in bulimic-type eating disorders. *Social Cognitive and Affective Neuroscience*, *11*(9), 1393–1401. https://doi.org/10.1093/scan/nsw049

Strigo, I. A., Matthews, S. C., Simmons, A. N., Oberndorfer, T., Klabunde, M., Reinhardt, L. E., & Kaye, W. H. (2013). Altered insula activation during pain anticipation in individuals recovered from anorexia nervosa: Evidence of interoceptive dysregulation. *The International Journal of Eating Disorders*, *46*(1), 23–33. https://doi.org/10.1002/eat.22045

Uher, R., Brammer, M. J., Murphy, T., Campbell, I. C., Ng, V. W., Williams, S. C. R., & Treasure, J. (2003). Recovery and chronicity in anorexia nervosa: Brain activity associated with differential outcomes. *Biological Psychiatry*, *54*(9), 934–942. https://doi.org/10.1016/S0006-3223(03)00172-0

Uher, R., Murphy, T., Brammer, M. J., Dalgleish, T., Phillips, M. L., Ng, V. W., Andrew, C. M., Williams, S. C. R., Campbell, I. C., & Treasure, J. (2004). Medial Prefrontal Cortex Activity Associated With Symptom Provocation in Eating Disorders. *American Journal of Psychiatry*, *161*(7), 1238–1246. https://doi.org/10.1176/appi.ajp.161.7.1238

Wierenga, C. E., Bischoff-Grethe, A., Berner, L. A., Simmons, A. N., Bailer, U., Paulus, M. P., & Kaye, W. H. (2020). Increased anticipatory brain response to pleasant touch in women remitted from bulimia nervosa. *Translational Psychiatry*, *10*, 236. https://doi.org/10.1038/s41398-020-00916-0

# Table S1.

Brain regions and Talairach coordinates for peaks showing convergence in activation when excluding two food addition related experiments during all anticipation tasks.

| Condition | Cluster’s anatomical location (BA) | Talairach coordinates | | | ALE (*10^-3^) | *Z* values | *p* values | Cluster size (mm^3^) |
| --- | --- | --- | --- | --- | --- | --- | --- | --- |
|  |  | x | y | z |  |  |  |  |
| *All anticipation* | | | | | | | | |
| DE > nDE* | Right putamen | 26 | -8 | 6 | 13.9 | 4.40 | 5.37E-06 | 672 |
|  |  | 26 | -12 | 8 | 13.5 | 4.30 | 8.36E-06 |  |

# Table S2.

Jackknife sensitivity analysis of anticipatory activation: DE > nDE (cluster in the right putamen).

| **Study** | **Contrast/condition** | **Contribution to cluster^a^** | **Jackknife sensitivity analysis^b^** | **Number of remaining clusters^c^** |
| --- | --- | --- | --- | --- |
| (Balodis et al., 2013) | Monetary win vs. neutral anticipation |  | X | 1 |
| (Balodis et al., 2013) | Monetary loss vs. neutral anticipation |  | X | 1 |
| (Berner et al., 2019) | Aversive breathing load anticipation | X |  | 0 |
| (Cowdrey et al., 2011) | Chocolate (pleasant food) anticipation |  | X | 1 |
| (Ehrlich et al., 2015) | Reward anticipation |  | X | 1 |
| (Gearhardt et al., 2011) | Pleasant vs. tasteless food anticipation |  | X | 1 |
| (Horndasch et al., 2018) | High calorie food anticipation |  | X | 1 |
| (Horndasch et al., 2018) | Low calorie food anticipation |  | X | 1 |
| (Kaye et al., 2010) | Food images anticipation |  | X | 1 |
| (Murao et al., 2017) | Monetary win vs. neutral anticipation |  | X | 1 |
| (Oberndorfer et al., 2013) | Food anticipation | X |  | 0 |
| (Schulte et al., 2019) | Highly processed vs. minimally processed food anticipation |  | X | 1 |
| (Strigo et al., 2013) | Pain anticipation |  | X | 1 |
| (Uher et al., 2003) | Food vs. non-food anticipation |  | X | 1 |
| (Uher et al., 2004) | Food vs. non-food anticipation |  | X | 1 |
| (Uher et al., 2004) | Aversive vs. neutral anticipation |  | X | 1 |
| (Wierenga et al., 2020) | Soft touch anticipation |  | X | 1 |

Notes. ^a^: whether each experiment contributes directly to the cluster in ALE meta-analysis; ^b^: the effects or each experiment removal on the results in the significant cluster; ^c^: the number of clusters remaining after leaving each experiment out.

# Table S3.

Jackknife sensitivity analysis of anticipatory deactivation: DE < nDE (cluster in the left inferior parietal lobule).

| **Experiment** | **Contrast/condition** | **Contribution to cluster^a^** | **Jackknife sensitivity analysis^b^** | **Number of remaining clusters^c^** |
| --- | --- | --- | --- | --- |
| (Balodis et al., 2013) | Monetary win vs. neutral anticipation |  | X | 1 |
| (Balodis et al., 2013) | Monetary loss vs. neutral anticipation |  | X | 1 |
| (Berner et al., 2018) | Aversive breathing load anticipation |  | X | 1 |
| (Brooks et al., 2011) | Food vs. non-food anticipation |  | X | 1 |
| (Horndasch et al., 2018) | Low calorie food anticipation |  | X | 1 |
| (Jiang et al., 2019) | Food odour anticipation |  | X | 1 |
| (Joos et al., 2011) | Food vs. non-food anticipation |  | X | 1 |
| (Oberndorfer et al., 2013) | Food anticipation |  | X | 1 |
| (Scaife et al., 2016) | Low calorie food anticipation | X | X | 1 |
| (Simon et al., 2016) | High vs. low food reward anticipation |  | X | 1 |
| (Strigo et al., 2013) | Pain anticipation |  | X | 1 |
| (Uher et al., 2003) | Food vs. non-food anticipation | X | X | 1 |
| (Uher et al., 2004) | Food vs. non-food anticipation | X |  | 0 |
| (Uher et al., 2004) | Aversive vs. neutral anticipation |  | X | 1 |

Notes. ^a^: whether each experiment contributes directly to the cluster in ALE meta-analysis; ^b^: the effects or each experiment removal on the results in the significant cluster; ^c^: the number of clusters remaining after leaving each experiment out.

# Figure S1.

ALE maps of the cluster showing group differences in activation during all types of anticipation after excluding two food addiction related experiments.


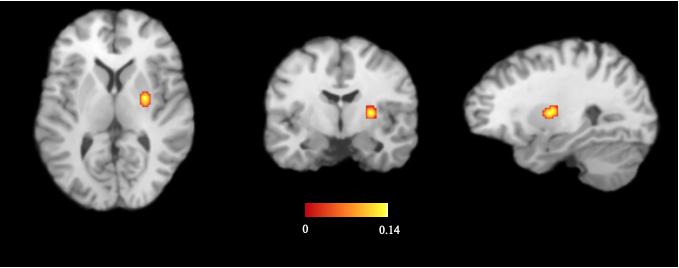

Supplement: Supplementary file 1 — Supplementary Material [file ERV-31-363-s001.docx]
